# Mitochondrial membrane depolarization enhances TRAIL-induced cell death in adult human granulosa tumor cells, KGN, through inhibition of *BIRC5*

**DOI:** 10.1186/s13048-018-0463-3

**Published:** 2018-10-16

**Authors:** Julie A. MacDonald, Niharika Kura, Carleigh Sussman, Dori C. Woods

**Affiliations:** Laboratory for Aging and Infertility Research, Northeastern University, Boston, MA 02115 USA

**Keywords:** Granulosa cell tumor, KGN, Mitochondria, Metabolism, TRAIL, BIRC5

## Abstract

**Background:**

Cellular metabolic changes that accompany malignant transformation have been heralded as hallmark features of cancer. However, metabolic signatures between neoplasms can be unique, allowing for distinctions in malignancy, invasion and chemoresistance between cancer types and subtypes. Mitochondria are central metabolic mediators, as cellular bioenergetics veers from oxidative phosphorylation to glycolysis. Herein, we evaluate the role of mitochondria in maintenance of cellular metabolism, proliferation, and survival in the adult granulosa tumor cell line, KGN, as well as three epithelial ovarian cancer cell lines to determine distinctions in specific features.

**Results:**

Notably, KGN cells were susceptible to TRAIL- and cisplatin-induced death following pretreatment with the metabolic inhibitor FCCP, but not oligomycin A. Collapse of mitochondrial membrane potential was found concomitant with cell death via apoptosis, independent from extrinsic canonical apoptotic routes. Rather, treatment with FCCP resulted in elevated cytochrome c release from mitochondria and decreased responsiveness to *BIRC5*. Following knockdown of *BIRC5*, mitochondrial membrane depolarization further sensitized KGN cells to induction of apoptosis via TRAIL.

**Conclusions:**

These results indicate an essential role, distinct from metabolism, for mitochondrial membrane potential in KGN cells to sense and respond to external mediators of apoptotic induction.

**Electronic supplementary material:**

The online version of this article (10.1186/s13048-018-0463-3) contains supplementary material, which is available to authorized users.

## Background

Among the subtypes of ovarian cancer, granulosa cell tumors (GCTs) of the ovary are relatively rare in occurrence, representing only approximately 2–5% of all ovarian neoplasms, yet represent the most common sex cord tumors [1]. This heterogeneous classification includes tumors derived from the stroma, rete ovarii, and developing ovarian follicles [1]. GCTs can be categorized as either adult (> 95%) or juvenile (< 5%) types, with adult GCTs (aGCTs) harboring a somatic missense mutation in the *FOXL2* gene, (*FOXL2*^C134W^), which is not present in the rarer juvenile subtype [2]. Arguably pathogenic, the mechanisms by which *FOXL2*^C134W^ drive tumorigenesis are not well characterized, but set this tumor type apart from not only the juvenile subtype, but ovarian cancers of epithelial origin as well [2–5]. Due to the relative paucity of information regarding the etiology, progression, and molecular properties of aGCTs given their comparatively infrequent manifestation, the prevailing therapeutic strategies used to treat aGCTs are derived from those used in treatment of more common epithelial ovarian cancers (EOCs), despite the distinct phenotype of aGCTs [6, 7]. Among the stark differences that distinguish GCTs from EOCs is the capacity of aGCTs to exhibit steroidogenesis associated with gonadotropin-responsiveness, categorizing aGCTs as endocrine tumors [4]. Consequently, unlike EOCs which can remain asymptomatic and evade detection until late-stage, the elevated estrogen synthesized by aGCTs initiates symptoms characteristic of estrogen excess, including menorrhagia and endometrial hyperplasia [8], resulting in earlier clinical detection than other ovarian cancers. However, recurrence is common and often delayed, with relapses occurring between 6– and 30–years from the initial diagnosis [9]. Accordingly, additional characterization of the aGCT response, as compared to EOCs, is an important consideration for the treatment of aGCTs.

Recent evidence from a number of cancer types highlights the cellular metabolic properties of tumor cells as a potential mediator of cancer growth and progression, metastasis, and evasion of chemotherapeutic-induced cell death. In cancer cells, a distinctive and near-universal trait is a metabolic switch in carbohydrate metabolism from oxidative phosphorylation (OXPHOS) to glycolysis [10]. While most normal cells generate ATP primarily from OXPHOS in aerobic conditions, tumor cells are more likely to synthesize ATP via conversion of glucose to lactate, and consequently exhibit a reduction in OXPHOS activity [11]. Although this metabolic shift to aerobic glycolysis at first glance appears to decrease energetic efficiency, it is believed the upregulation of glycolysis is a key step in carcinogenesis, providing a growth advantage to malignant cells capable of surviving a hypoxic microenvironment [12]. Malignant transformation is known to be associated with other alterations in mitochondrial function as well. For example, in normal cells a high mitochondrial membrane potential (Δψ_m_) reflects mitochondrial respiration and generation of ATP, whereas mitochondria in cancer cells demonstrate an aberrant Δψ_m_ [13]. These types of alterations in mitochondrial function specifically in cancer cells have raised prospects for therapeutic targeting of mitochondria in cancer cells. Notably, metabolic profiles defining OXPHOS vs glycolytic status for aGCT cells, as well as EOCs, have yet to be well- reported [14–17].

We have previously reported on the efficacy of the naturally occurring cytokine, tumor necrosis factor-related apoptosis-inducing ligand (TRAIL), to promote cell death in aGCTs under specific conditions [18, 19]. This is of particular interest, as evidence from primary cultured ovarian granulosa cells have demonstrated that under normal conditions, healthy granulosa cells are inherently resistant to TRAIL-induced apoptosis [20]. However, while treatment of aGCTs with TRAIL in combination with therapeutic agents such as cisplatin or proteasome inhibitors enhances TRAIL-induced cell death, this action is mediated largely through upregulation of death receptors (e.g., DR4 or DR5), and downstream effectors, such as p53, are not impacted [18, 19]. DR4 and DR5 are members of the tumor necrosis factor receptor (TNFR) superfamily [21], and are characterized by multiple cysteine-rich domains present within the extracellular domain, in addition to an intracellular death domain (DD) [22]. Following activation through TRAIL-binding, the DD links with Fas-associated death domain (FADD) protein, resulting in the formation of the apoptosis-inducing signaling complex (DISC) [23], which subsequently leads to activation of caspase-8, which cleaves bid and triggers the release of cytochrome c from the mitochondria into the cytosol [24], triggering the caspase cascade that ultimately results in cellular apoptosis. However, the ability of TRAIL to readily induce apoptosis is diminished, as the TRAIL-induced caspase cascade is opposed by pro-survival proteins, namely the inhibitor of apoptosis (IAP) family, which includes Survivin, also called baculoviral inhibitor of apoptosis repeat-containing 5 (BIRC5). Notably, BIRC5 is a potent inhibitor of caspase activation, and when present, actively promotes cell survival through inhibition of caspase-induced apoptosis. Moreover, and perhaps not surprisingly, BIRC5 levels are elevated in tumors as compared to normal tissues [25]. Additionally, BIRC5 is thought to play an important role in mediating aerobic glycolysis in cancer cells through suppression of OXPHOS, which occurs, at least in part, through inhibition of respiratory complex-I [26].

The disruption of mitochondria-driven metabolism, as well as mitochondria-mediated pro-apoptotic cascades, has been well-characterized in many models of cancer, although not yet including aGCTs. A better understanding of the molecular characteristics of aGCT metabolism, as well as the response to traditional therapeutics, could serve to inform novel therapies which specifically target aGCTs. Herein, we evaluated the metabolic profile of three human EOC cell lines, SKOV3, OVCAR3, and Kuramochi, as well as KGN, a well-established human aGCT cell line heterozygous for the *FOXL2*^C134W^ mutation. Through the use of metabolic inhibitors, we investigated the role of KGN metabolism in survival following treatment with either TRAIL or cisplatin, and determined an essential role for Δψm in the opposition of pro-apoptotic signals.

## Results

### Metabolism of ovarian cancer cell lines and impact of metabolic inhibitors on adult granulosa tumor cell line (KGN) culture viability

**Fig. 1:**
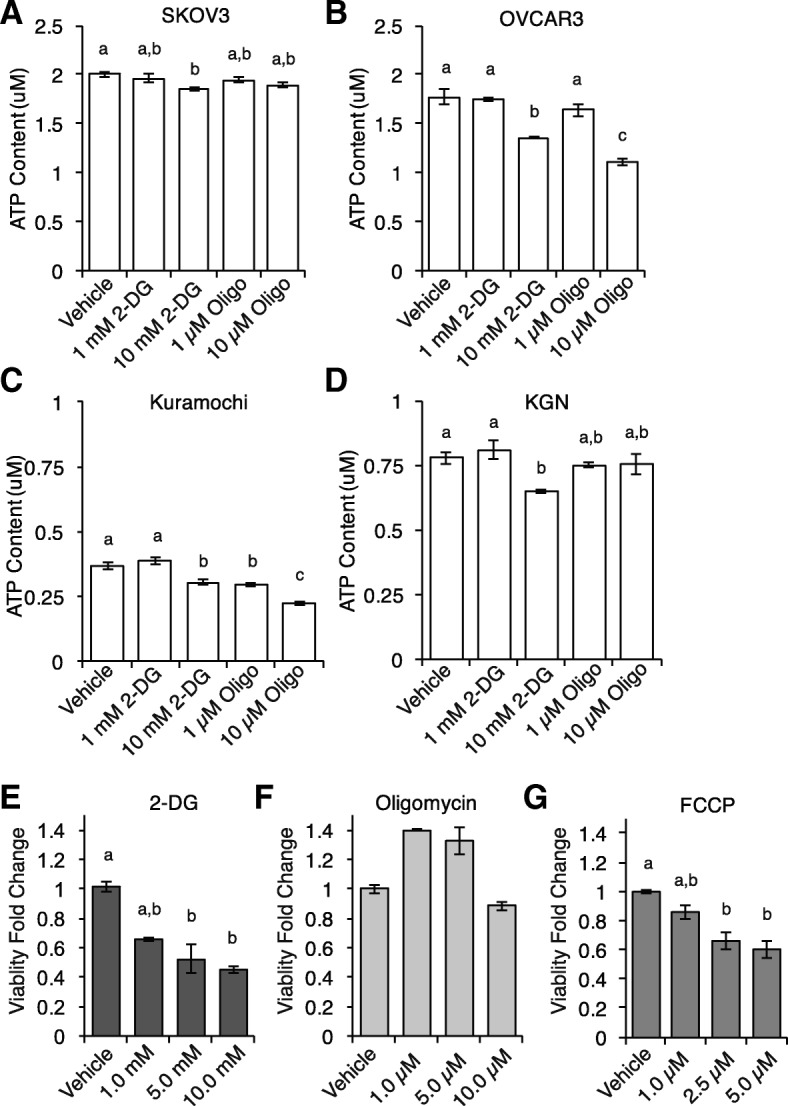
Metabolism of ovarian cancer cell lines and impact of metabolic inhibitors on adult granulosa tumor cell line (KGN) culture viability. Dynamics of metabolism of ovarian cancer cell lines was analyzed through disruption of both glycolysis and oxidative phosphorylation (OXPHOS). Total cellular ATP content was analyzed following 6-h treatments with glycolysis inhibitor 2-deoxy-d-glucose (2-DG) or OXPHOS inhibitor, oligomycin A (Oligo), where indicated conditions annotated with different letters indicates statistical significance between conditions of at least *P* < 0.05. **a** SKOV3 cells had significantly reduced total cellular ATP following treatment with 10 mM 2-DG. **b** OVCAR3 cells and (**c**) Kuramochi cells decreased in ATP content following treatment with high doses of both 2-DG and Oligo. **d** KGN cells had a significant decrease in total cellular ATP levels after treatment with 10 mM 2-DG. **e** Treatment with 5 mM and 10 mM 2-DG over 24 h significantly decreased KGN culture viability, as measured by MTS assay. **f** Treatment with oligomycin A for 24 showed no changes in KGN culture viability, whereas treatment with the respiratory chain uncoupler carbonyl cyanide-*4*-(trifluoromethoxy)phenylhydrazone (FCCP; **g**) significantly decreased culture viability at both 2.5 μM and 5.0 μM after 24 h

Epithelial ovarian cancer cell lines assayed for total cellular ATP content following a 6-h incubation with glycolysis inhibitor, 2-deoxy-d-glucose (2-DG), or oxidative phosphorylation inhibitor, oligomycin A, were heterogeneous in metabolic profiles (Fig. 1). SKOV3 cells demonstrated reduced ATP content as compared to vehicle treated cells following a high dose of 2-DG, indicating glycolytic metabolism (Vehicle: 1.99 ± 0.03 μM; 10 mM 2-DG: 1.85 ± 0.02 μM, *P* < 0.05; Fig. 1a). Treatment with either inhibitor resulted in a reduction in ATP content in both OVCAR3 and Kuramochi (OVCAR3: Vehicle: 1.77 ± 0.08 μM; 10 mM 2-DG: 1.36 ± 0.01, *P* < 0.01; 10 μM Oligomycin: 1.11 ± 0.03, *P* < 0.01, *n* = 3; Fig. 1b. Kuramochi: Vehicle: 0.37 ± 0.01 μM; 10 mM 2-DG: 0.30 ± 0.01, *P* < 0.01; 10 μM Oligomycin: 0.22 ± 0.01, *P* < 0.01, *n* = 3; Fig. 1c). In KGN, total cellular ATP content was significantly decreased as compared to vehicle-treated controls following a 6-h incubation with the glycolysis inhibitor 2-DG (Vehicle: 0.78 ± 0.02 μM; 10 mM 2-DG: 0.65 ± 0.01 μM, *P* < 0.05, *n* = 3), with no change in samples treated with the oxidative phosphorylation inhibitor oligomycin A (10 μM Oligomycin A; 0.75 ± 0.04 μM, *n* = 3), consistent with glycolytic metabolism (Fig. 1d).

Culture viability of KGN cells was assayed using a MTS assay following 24-h treatments with metabolic inhibitors. Following treatment with 2-DG (Fig. 1e), viability of KGN cells was significantly reduced as compared to vehicle controls at both 5 mM and 10 mM doses (5 mM: 0.52 ± 0.12-fold change, *P* < 0.05; 10 mM: 0.45 ± 0.06-fold change, *P* < 0.01, *n* = 3). However, after 24-h treatments with oligomycin A, viability remained unchanged (*n* = 9; Fig. 1f). Interestingly, treatment with carbonyl cyanide-*4*-(trifluoromethoxy)phenylhydrazone (FCCP; Fig. 1g), a metabolic inhibitor which uncouples Δψ_m_ from ATP synthesis [27], also led to a significant reduction in culture viability following 24-h treatments at 2.5 μm and 5.0 μM as compared to vehicle controls (2.5 μM: 0.66 ± 0.06-fold change, *P* < 0.01; 5.0 μM: 0.60 ± 0.06-fold change, *P* < 0.01, *n* = 9), of note due to the otherwise glycolytic nature of the cells.

### Metabolic inhibitors sensitize KGN cells to cytotoxicity via TRAIL or cisplatin

Pretreatment with metabolic inhibitors FCCP or oligomycin A sensitized KGN cells, which are otherwise predominately resistant, to cell death induction by TRAIL or the chemotherapeutic cisplatin (Table 1). Following pretreatment with 5.0 μM FCCP, KGN cells treated with either TRAIL (50 and 100 ng mL^− 1^; Table 1A) or cisplatin (1 and 10 μM; Table 1B), demonstrated reduced cell viability as compared to vehicle-treated controls. Additionally, oligomycin A pretreatment sensitized KGN cells to significant decreases in culture viability following treatment with 100 ng ml^− 1^ TRAIL (Table 1C) or 10 μM cisplatin (Table 1D). When these assays were performed in the ovarian epithelial carcinoma cell lines SKOV3 (Table 2), Kuramochi (Table 3), and OVCAR3 (Table 4), the trend of metabolic inhibition increasing sensitivity to cytotoxic agents was repeated, but was inconsistent between the cell lines. In SKOV3 cultures, cell viability was reduced following oligomycin A pretreatment, and subsequent treatment with either TRAIL or cisplatin (Table 2C-D), whereas pretreatment with FCCP sensitized cells to TRAIL (Table 2A), but not cisplatin (Table 2B). FCCP treatment also resulted in decreased viability in Kuramochi when treated in combination with TRAIL (Table 3A), but not with cisplatin (Table 3B). Interestingly, when treated with oligomycin A (Table 3C-D), Kuramochi cultures increased in viability, with the only significant decrease in cisplatin treated cells observed after pretreatment with 2.5 μM of oligomycin A (Table 3D). The OVCAR3 cell line was previously reported as TRAIL-sensitive [28], which was repeated herein (Table 4A, C), as well as a demonstrated sensitivity to cisplatin without metabolic inhibition (Table 4B, D). However, pretreatment with both FCCP and oligomycin further sensitized OVCAR3 cells to cisplatin treatment (Table 4B,D).

**Table 1:** Metabolic inhibition sensitize KGN cultures to cytotoxicity via TRAIL and cisplatin

A		TRAIL			
		Untreated	50 ng/mL	100 ng/mL	ANOVA
FCCP (μM)	Vehicle	1.00 ± 0.01^a^	0.83 ± 0.06^a*^	0.97 ± 0.04^a^	0.0206
	1.0	0.94 ± 0.02^a^	0.84 ± 0.06^a,b^	0.85 ± 0.04^a,b^	0.1921
	2.5	0.74 ± 0.05^b^	0.74 ± 0.04^b^	0.74 ± 0.04^b^	0.9967
	5.0	0.68 ± 0.05^b^	0.44 ± 0.02^c^	0.49 ± 0.06^c*^	0.0327
ANOVA		2.30E-06	2.82E-06	7.48E-08	
B		Cisplatin			
		Untreated	1 μM	10 μM	ANOVA
FCCP (μM)	Vehicle	1.00 ± 0.10^a^	1.21 ± 0.16^a^	0.98 ± 0.06^a^	0.3321
	1.0	0.93 ± 0.07^a^	1.03 ± 0.12^a^	0.81 ± 0.03^b^	0.1766
	2.5	0.91 ± 0.03^a^	0.97 ± 0.16^a^	0.79 ± 0.01^b^	0.4927
	5.0	0.79 ± 0.03^a^	0.89 ± 0.08^a^	0.59 ± 0.03^c*^	0.0155
ANOVA		0.1798	0.427	0.0005	
C		TRAIL			
		Untreated	50 ng/mL	100 ng/mL	ANOVA
Oligomycin (μM)	Vehicle	1.00 ± 0.05^a^	1.15 ± 0.08^a^	0.96 ± 0.02^a^	0.1381
	1.0	0.99 ± 0.08^a^	1.01 ± 0.03^a^	0.81 ± 0.01^b^	0.0511
	2.5	1.00 ± 0.07^a^	0.97 ± 0.01^a^	0.82 ± 0.02^b*^	0.0368
	5.0	1.06 ± 0.02^a^	0.97 ± 0.03^a^	0.83 ± 0.02^b*^	0.0013
ANOVA		0.8191	0.0827	0.0013	
D		Cisplatin			
		Untreated	1 μM	10 μM	ANOVA
Oligomycin (μM)	Vehicle	1.00 ± 0.05^a^	0.85 ± 0.04^a^	0.65 ± 0.07^a^	0.0144
	1.0	1.08 ± 0.06^a^	1.03 ± 0.08^a^	0.84 ± 0.02^b^	0.0548
	2.5	1.03 ± 0.03^a^	1.05 ± 0.07^a^	0.83 ± 0.02^b*^	0.0293
	5.0	0.98 ± 0.02^a^	0.94 ± 0.03^a^	0.77 ± 0.06^b*^	0.0206
ANOVA		0.4821	0.161	0.0802	

Resistance of KGN cultures to cell death inducing agents TRAIL or cisplatin was analyzed through inhibition of OXPHOS using oligomycin A and a mitochondrial membrane potential uncoupler, FCCP, using MTS viability assays. Results are presented as fold change to vehicle treated control cultures with differing letters indicating significant (*p* < 0.05) changes in viability with treatment of inhibitor, and asterisks (* *p* < 0.05) indicating significant changes in viability with treatment of cytotoxic agent. (**A**) Sequential treatment with FCCP and TRAIL resulted in decreases in culture viability over control samples. (**B**) Culture viability also decreased with FCCP treatment prior to addition of cisplatin. (**C**) Oligomycin A pretreatment sensitized KGN cells to both TRAIL and (**D**) cisplatin

**Table 2:** Metabolic inhibitors sensitize SKOV3 cultures to cytotoxicity via TRAIL or cisplatin

A		TRAIL			
		Untreated	50 ng/mL	100 ng/mL	ANOVA
FCCP (μM)	Vehicle	1.00 ± 0.02^a^	0.90 ± 0.06^a^	0.93 ± 0.02^a^	0.1901
	1.0	0.98 ± 0.04^a^	0.85 ± 0.04^a*^	0.90 ± 0.02^a^	0.0448
	2.5	0.94 ± 0.03^a^	0.75 ± 0.04^a**^	0.79 ± 0.01^b**^	0.0011
	5.0	0.91 ± 0.02^a^	0.69 ± 0.03^b**^	0.72 ± 0.02^b**^	7.16E-05
ANOVA		0.1335	0.0143	1.28E-06	
B		Cisplatin			
		Untreated	1 μM	10 μM	ANOVA
FCCP (μM)	Vehicle	1.00 ± 0.01^a^	0.95 ± 0.07^a^	1.07 ± 0.04^a^	0.2609
	1.0	0.96 ± 0.04^a,b^	0.85 ± 0.03^a^	0.99 ± 0.04^a^	0.1036
	2.5	0.85 ± 0.00^b,c^	0.91 ± 0.05^a^	0.95 ± 0.04^a^	0.2008
	5.0	0.80 ± 0.02^c^	0.78 ± 0.04^a^	0.88 ± 0.02^b^	0.1185
ANOVA		0.0012	0.1549	0.0384	
C		TRAIL			
		Untreated	50 ng/mL	100 ng/mL	ANOVA
Oligomycin (μM)	Vehicle	1.0 ± 0.04^a^	0.97 ± 0.04^a^	0.95 ± 0.02^a^	0.5931
	1.0	0.90 ± 0.01^a^	0.85 ± 0.01^a^	0.80 ± 0.01^b**^	0.006
	2.5	0.96 ± 0.04^a^	0.82 ± 0.05^a^	0.81 ± 0.00^b^	0.0483
	5.0	0.86 ± 0.03^a^	0.82 ± 0.08^a^	0.84 ± 0.04^b^	0.9093
ANOVA		0.0507	0.2121	0.0062	
Cisplatin					
		Untreated	1 μM	10 μM	ANOVA
Oligomycin (μM)	Vehicle	1.00 ± 0.03^a^	0.88 ± 0.09^a^	0.94 ± 0.02^a^	0.3459
	1.0	0.92 ± 0.02^a^	0.85 ± 0.01^a*^	0.84 ± 0.01^b*^	0.0129
	2.5	0.91 ± 0.02^b^	0.84 ± 0.03^a^	0.89 ± 0.01^a^	0.1578
	5.0	0.91 ± 0.00^b^	0.88 ± 0.03^a^	0.84 ± 0.03^b^	0.2232
ANOVA		0.0279	0.9114	0.0135	

Resistance of SKOV3 cultures to cell death inducing agents TRAIL or cisplatin was analyzed through inhibition of OXPHOS using oligomycin A and a mitochondrial membrane potential uncoupler, FCCP, using MTS viability assays. Results are presented as fold change to vehicle treated control cultures with differing letters indicating significant (*p* < 0.05) changes in viability with treatment of inhibitor, and asterisks indicating significant (* *p* < 0.05; ** *p* < 0.01) changes in viability with treatment of cytotoxic agent (**A**) Pretreatment with FCCP sensitized SKOV3 cultures to TRAIL, (**B**) but not cisplatin. (**C**) Culture viability was decreased with oligomycin A treatment prior to TRAIL, (**D**) as well as cisplatin

**Table 3:** FCCP pretreatment sensitize Kuramochi cultures to TRAIL and oligomycin A increases baseline Kuramochi culture viability

A		TRAIL			
		Untreated	50 ng/mL	100 ng/mL	ANOVA
FCCP (μM)	Vehicle	1.00 ± 0.08^a^	1.00 ± 0.06^a^	0.94 ± 0.07^a^	0.7702
	1.0	0.89 ± 0.03^a,b^	0.72 ± 0.03^b*^	0.74 ± 0.03^a*^	0.009
	2.5	0.74 ± 0.03^b,c^	0.67 ± 0.02^b^	0.60 ± 0.02^b*^	0.0233
	5.0	0.66 ± 0.02^c^	0.58 ± 0.06^b^	0.57 ± 0.05^b^	0.3392
ANOVA		0.0035	0.0009	0.002	
B		Cisplatin			
		Untreated	1 μM	10 μM	ANOVA
FCCP (μM)	Vehicle	1.00 ± 0.06^a^	0.97 ± 0.13^a^	1.11 ± 0.02^a^	0.5124
	1.0	0.89 ± 0.01^a^	0.82 ± 0.10^a^	0.85 ± 0.01^b^	0.6946
	2.5	0.99 ± 0.09^a^	0.78 ± 0.08^a^	0.75 ± 0.01^c^	0.0941
	5.0	0.90 ± 0.01^a^	0.74 ± 0.10^a^	0.78 ± 0.04^b,c^	0.23
ANOVA		0.3707	0.4635	7.78E-06	
C		TRAIL			
		Untreated	50 ng/mL	100 ng/mL	ANOVA
Oligomycin (μM)	Vehicle	1.00 ± 0.02^a^	0.96 ± 0.14^a^	1.08 ± 0.03^a^	0.5539
	1.0	1.37 ± 0.00^b^	1.15 ± 0.07^a^	1.26 ± 0.07^a^	0.093
	2.5	1.38 ± 0.12^b^	1.14 ± 0.15^a^	1.31 ± 0.04^a^	0.353
	5.0	1.36 ± 0.05^b^	1.19 ± 0.16^a^	1.25 ± 0.05^a^	0.535
ANOVA		0.0082	0.6272	0.0503	
D		Cisplatin			
		Untreated	1 μM	10 μM	ANOVA
Oligomycin (μM)	Vehicle	1.00 ± 0.01^a^	0.99 ± 0.01^a^	1.01 ± 0.02^a^	0.6714
	1.0	1.46 ± 0.18^a,b^	1.29 ± 0.16^a^	1.62 ± 0.03^b^	0.3404
	2.5	1.83 ± 0.06^b^	1.21 ± 0.04^a**^	1.55 ± 0.09^b,c*^	0.0014
	5.0	1.23 ± 0.08^a^	1.10 ± 0.04^a^	1.13 ± 0.02^a^	0.2911
ANOVA		0.003	0.1401	4.23E-05	

Resistance of Kuramochi cultures to cell death inducing agents TRAIL or cisplatin was analyzed through inhibition of OXPHOS using oligomycin A and a mitochondrial membrane potential uncoupler, FCCP, using MTS viability assays. Results are presented as fold change to vehicle treated control cultures with differing letters indicating significant (*p* < 0.05) changes in viability with treatment of inhibitor, and asterisks indicating significant (* *p* < 0.05; ** *p* < 0.01) changes in viability with treatment of cytotoxic agent. (A) Pretreatment with FCCP resulted in reduced culture viability following treatment with TRAIL, (B) but not cisplatin. (C) Oligomycin treatment increased culture viability, when compared to vehicle, but did not sensitize Kuramochi cultures to TRAIL. (D) Pretreatment with 2.5 μM oligomycin A sensitized Kuramochi cultures to decrease viability following cisplatin treatment

**Table 4:** FCCP and oligomycin pretreatment sensitize OVCAR3 cultures to cisplatin treatment

A		TRAIL			
		Untreated	50 ng/mL	100 ng/mL	ANOVA
FCCP (μM)	Vehicle	1.00 ± 0.12^a^	0.12 ± 0.01^a,b,c*^	0.07 ± 0.02^a*^	0.0001
	1.0	0.98 ± 0.09^a^	0.21 ± 0.06^a,b*^	0.06 ± 0.01^a*^	0.0001
	2.5	0.77 ± 0.08^a^	0.06 ± 0.05^a,b,c*^	0.00 ± 0.02^a,b*^	0.0001
	5.0	0.70 ± 0.03^a^	0.01 ± 0.02^a,c*^	−0.02 ± 0.01^b*^	5.89E-07
ANOVA		0.0929	0.0436	0.0087	
B		Cisplatin			
		Untreated	1 μM	10 μM	ANOVA
FCCP (μM)	Vehicle	1.00 ± 0.07^a^	1.16 ± 0.03^a^	0.79 ± 0.03^a*^	0.0052
	1.0	0.84 ± 0.02^a,b^	0.89 ± 0.02^b*^	0.60 ± 0.01^b*^	8.79E-05
	2.5	0.70 ± 0.01^b,c^	0.79 ± 0.02^b,c*^	0.52 ± 0.01^b,c*^	7.98E-05
	5.0	0.54 ± 0.03^c^	0.57 ± 0.02^d^	0.40 ± 0.00^d*^	0.0015
ANOVA		0.0002	1.22E-06	2.47E-06	
C		TRAIL			
		Untreated	50 ng/mL	100 ng/mL	ANOVA
Oligomycin (μM)	Vehicle	1.00 ± 0.01^a^	0.09 ± 0.02^a*^	0.14 ± 0.01^a*^	2.90E-08
	1.0	0.94 ± 0.03^a^	0.01 ± 0.00^a*^	0.07 ± 0.00^b*^	6.04E-05
	2.5	0.89 ± 0.05^a^	0.01 ± 0.02^a*^	0.08 ± 0.01^b*^	3.19E-06
	5.0	0.78 ± 0.08^a^	0.06 ± 0.02^a*^	0.09 ± 0.01^a*^	5.10E-05
ANOVA		0.0988	0.0629	0.0091	
D		Cisplatin			
		Untreated	1 μM	10 μM	ANOVA
Oligomycin (μM)	Vehicle	1.00 ± 0.02^a^	1.10 ± 0.05^a^	0.91 ± 0.03^a*^	0.0385
	1.0	0.91 ± 0.04^a^	1.09 ± 0.03^a*^	0.80 ± 0.03^a^	0.0031
	2.5	0.92 ± 0.02^a^	1.08 ± 0..02^a^	0.83 ± 0.05^a^	0.1875
	5.0	0.90 ± 0.05^a^	0.99 ± 0.03^a^	0.87 ± 0.02^a^	0.0864
ANOVA		0.2729	0.1875	0.2136	

Resistance of OVCAR3 cultures to cell death inducing agents TRAIL or cisplatin was analyzed through inhibition of OXPHOS using oligomycin A and a mitochondrial membrane potential uncoupler, FCCP, using MTS viability assays. Results are presented as fold change to vehicle treated control cultures with differing letters indicating significant (*p* < 0.05) changes in viability with treatment of inhibitor, and asterisks indicating significant (* *p* < 0.05) changes in viability with treatment of cytotoxic agent. (**A,C**) OVCAR3 cultures are sensitive to cell-death via TRAIL. (**B,D**) OVCAR3 cultures were significantly sensitive to treatment with cisplatin, however pretreatment with both FCCP and oligomycin further decreased culture viability

### Combinatorial treatment with FCCP and TRAIL induces apoptosis and disrupts both cellular metabolism and Δψ_m_

**Fig. 2:**
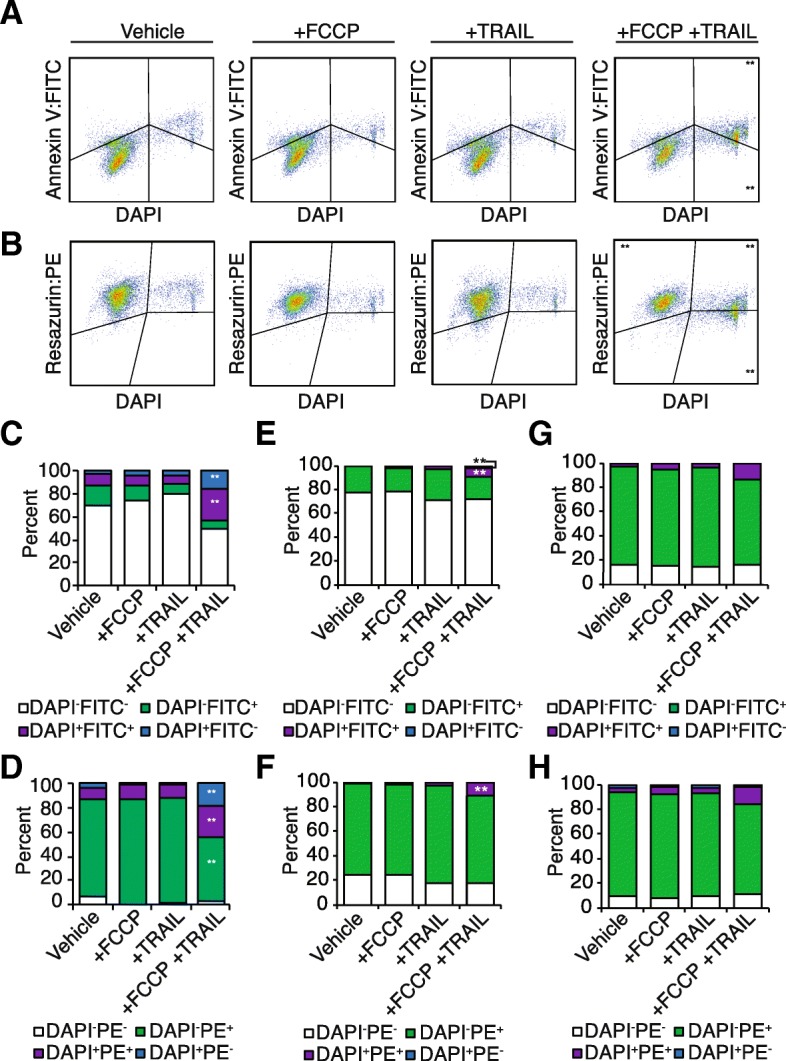
Dual treatment with FCCP and TRAIL induces apoptosis and disrupts cellular metabolism. Dual treated cells were analyzed for extracellular expression of phosphatidylserine by antibody labeling with Annexin V (AnnexinV:FITC), the fluorescent metabolic dye resazurin (Resazurin:PE), and viability probe DAPI to determine apoptotic induction. **a** Co-labeling with AnnexinV:FITC and DAPI indicated high viability in control wells, with significant increases in both apoptotic cells (DAPI^+^FITC^+^; purple) as well as nonviable cells (DAPI^+^FITC^−^; blue) following both FCCP and TRAIL (**c**: *n* = 3 ANOVA *p* < 0.001**). **b** The increase in DAPI^+^ events was also present when evaluating changes in global cellular metabolism, indicative of both respiring non-viable cells, as well as non-respiring non-viable cells. Additionally, there was a significant decrease in viable and metabolically active cells (DAPI^−^PE^+^; green) only after dual treatment with FCCP and TRAIL (**d**: *n* = 3 ANOVA *p* < 0.001**.) **e** In Kuramochi cultures, there was significant increases in both apoptotic cells as well as nonviable cells following treatment with both FCCP and TRAIL in the Kuramochi cell line (*n* = 3 ANOVA *p* < 0.001**), as well as (**f**) nonviable respiring cells (*n* = 3, ANOVA *p* < 0.001**). **g**, **h** SKOV3 cultures had no significant changes in apoptotic induction or metabolic modulation following treatment with FCCP and TRAIL

Cells treated with the combination of FCCP and TRAIL were analyzed for extracellular expression of phosphatidylserine by antibody labeling with Annexin V to determine apoptotic induction (Fig. 2). Co-labeling with DAPI (Fig. 2a) indicated a high culture viability in vehicle control and single treatment wells (~ 85–90% viable, similar to that observed utilizing the MTS assays), with significant increases in both apoptotic cells (DAPI^+^/FITC^+^; purple) as well as nonviable cells (DAPI^+^/FITC^−^; blue) following both FCCP and TRAIL (Fig. 2c). DAPI-positive events also increased when evaluating changes in global cellular metabolism utilizing the dye resazurin, which fluoresces when reduced, indicative of actively respiring cells [29] (Fig. 2b, d). These trends also occurred in the ovarian epithelial carcinoma line Kuramochi (Fig. 2e, f), but there were no statistical differences in apoptotic induction seen in SKOV3 cells following treatment with FCCP and TRAIL (Fig. 2g, h).

**Fig. 3:**
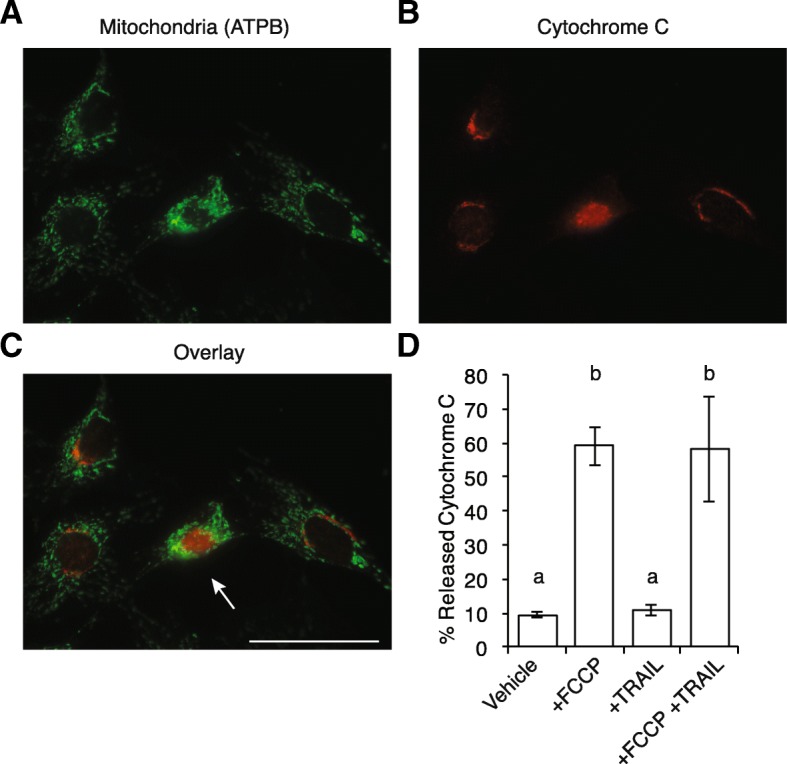
Cytosolic localization of cytochrome c following dual treatment with FCCP and TRAIL indicates induction of apoptosis in KGN cells. Cytochrome c release was assayed via immunocytochemistry to detect induction of apoptosis. Following treatment with FCCP and/or TRAIL, cells were fixed and immunolabeled with antibodies against (**a**) the mitochondrial marker ATPB (green) and (**b**) cytochrome C (red). In addition, cells were also labeled with DAPI to visualize the cell nuclei and establish random fields of view for imaging. **c** Representative image indicating a cell (white arrow) scored as having released cytochrome C due to the loss of colocalization with ATPB. **d** Vehicle treated and TRAIL treated cells contained a small percentage of cells with released cytochrome C whereas both FCCP treated and FCCP and TRAIL treated cells showed significant increases in the percentage of cells exhibiting released cytochrome C (59.05 ± 5.39%, *P* < 0.05, 58.17 ± 15.65%, *P* < 0.05, respectively. Differing letters indicate significance). Scale bar indicates 50 μm

To confirm induction of apoptosis in KGN cells following treatment with FCCP and TRAIL, cells were fixed and evaluated for cytochrome c release via immunocytochemistry (Fig. 3). ATPB was used as an identifying marker for mitochondria (Fig. 3a, c), and cells were scored as having released cytochrome c via loss of colocalization with ATPB (Fig. 3b, c). In vehicle treated and TRAIL treated cells there was a low percentage of cells scored as having released cytochrome C (9.49 ± 0.98%, 10.79 ± 1.60%, respectively), but in both FCCP treated and dual treated cells there were significant increases in the population of cells with diffuse cytochrome c localization (59.05 ± 5.39%, *P* < 0.05, 58.17 ± 15.65%, *P* < 0.05, respectively, Fig. 3d).

**Fig. 4:**
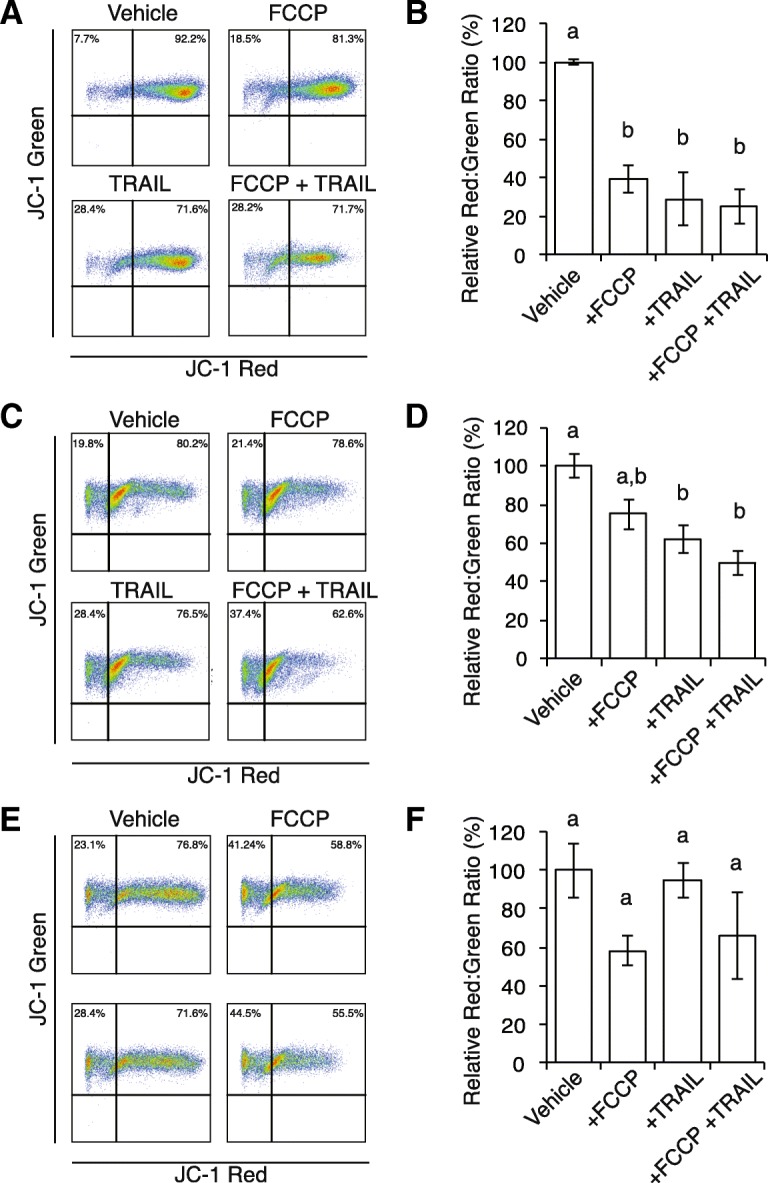
KGN and SKOV3, but not Kuramochi, cells exhibit decreased mitochondrial membrane potential (Δψ_m_) following dual treatment with FCCP and TRAIL. Flow cytometry was used to detect changes in Δψ_m_ using the fluorescent dye, JC-1. **a** Δψ_m_ independent JC-1 monomers fluoresce at ~ 525 nm (green) throughout the cell, while Δψ_m_ dependent JC-1 polymers form following accumulation within mitochondria and fluoresce at ~ 595 nm (orange-red), allowing quantitation of Δψ_m_ as the JC-1 red:JC-1 green ratio, representative plots of KGN culture. **b** Quantitation of the JC-1 red:JC-1 green ratio in KGN culture exhibited a decreased Δψ_m_ across all conditions tested (FCCP: 39 ± 7% relative to vehicle treated cells, with differing letters indicating statistical significance. **c**, **d** In SKOV3 cells FCCP treatment did not significantly decrease the JC-1 red:JC-1 green ratio, however single treatment with TRAIL decreased Δψ_m_, and was further decreased following treatment with both FCCP and TRAIL (**e**, **f**) In Kuramochi cells, no significant changes in Δψ_m_ were observed under the same conditions

As an additional characterization of apoptosis, Δψ_m_ was measured as the relative fluorescence of the Δψ_m_ dependent dye: JC-1. Following Δψ_m_-dependent accumulation within mitochondria, JC-1 J-aggregates fluoresce at ~ 590 nm (orange-red), whereas background fluorescence of JC-1 monomers fluoresce at ~ 525 nm (green), and the proportion of such populations (JC-1 red:JC-1 green ratio) analyzed via flow cytometry (Fig. 4). Not surprisingly, KGN cells (Fig. 4a) treated with the Δψ_m_ uncoupler FCCP had a significant decrease (Fig. 4b; 39 ± 7% relative to vehicle treated cells, *n* = 3, *P* < 0.01**)** in the JC-1 red:JC-1 green ratio, however, this effect was also in cells treated with TRAIL alone (29 ± 14%, *n* = 3, *P* < 0.01), as well as in combination (FCCP and TRAIL together: 25 ± 9%, *n* = 3, *P* < 0.01), indicative of a collapse in Δψ_m_ associated with apoptotic induction. In SKOV3 cells FCCP treatment alone did not significantly decrease the JC-1 red:JC-1 green ratio (Fig. 4c, d), however single treatment with TRAIL did decrease Δψ_m_ (62 ± 7% relative to vehicle, *n* = 3, *P* < 0.05) and was further decreased following dual treatment (50 ± 7%, *n* = 3, *P* < 0.01). No significant changes in Δψ_m_ were observed in Kuramochi cells under the same conditions (Fig. 4e, f; ANOVA *P* = 0.19). These data collectively indicate distinct cell-specific responses to alterations in Δψ_m_.

### Inhibition of BIRC5 sensitizes KGN cells to TRAIL-induced apoptosis following mitochondrial membrane depolarization

**Fig. 5:**
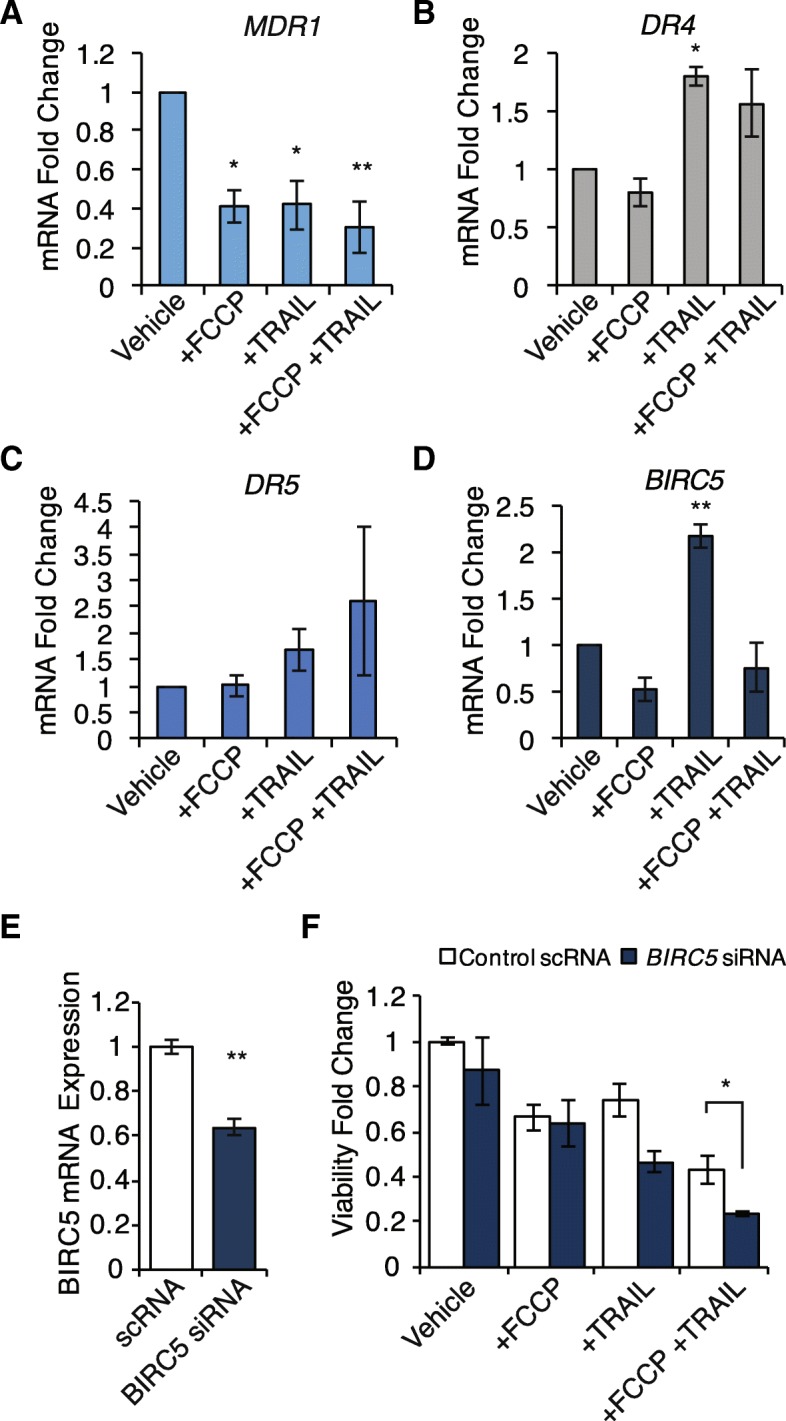
Inhibition of BIRC5 sensitizes KGN cells to TRAIL-induced apoptosis following mitochondrial membrane depolarization. Gene expression analysis of *MDR1, DR4, DR5*, and *BIRC5* following treatment with FCCP and/or TRAIL. **a** Expression of the ATP dependent efflux pump, *MDR1,* which conveys multidrug resistance, was downregulated following all treatment conditions, asterisks indicate significance with regard to vehicle treated cells- *P* < 0.05* and *P* < 0.01**. **b** *DR4* was significantly upregulated by treatment with TRAIL alone, but the secondary receptor for TRAIL, *DR5* (**c**), showed no change in expression (*P* = 0.42*)*. **d** The negative regulator of apoptosis, *BIRC5*, was significantly upregulated following treatment with TRAIL, however when pretreated with FCCP this response was lost. **e** A knockdown of *BIRC5* (64.0 ± 3.3% mRNA expression, 48 h post treatment, *n* = 3, *P* < 0.01**) prior to treatment (**f**) led to a significant enhancement of apoptosis induction by dual treatment with FCCP and TRAIL

Following treatment, KGN cells were collected for gene expression analysis of *MDR1, DR4, DR5, and BIRC5* (Fig. 5). The mRNA expression of the ATP dependent efflux pump, *MDR1* was significantly downregulated following all treatment conditions (Fig. 5a, FCCP: 0.41 ± 0.08- fold change, *n* = 3, *P* < 0.05; TRAIL: 0.42 ± 0.12- fold change, *n* = 3, *P* < 0.05; FCCP and TRAIL: 0.30 ± 0.13- fold change, *n* = 3, *P* < 0.01). Of the primary receptors for extrinsic activation of apoptosis by TRAIL, *DR4* (Fig. 5b) and *DR5* (Fig. 5c), only *DR4* mRNA was significantly upregulated by treatment with TRAIL alone (1.79 ± 0.09-fold change, *n* = 3, *P* < 0.05). On the other hand, *BIRC5*, a negative regulator of apoptosis, was significantly upregulated following treatment with TRAIL (Fig. 5d; 2.17 ± 0.14-fold change, *n* = 3, *P* < 0.01), and this upregulation was prevented when pretreated with FCCP (0.76 ± 0.25-fold change, *n* = 3). To determine if loss of this negative regulator would additionally sensitize KGN to apoptosis, we completed a knockdown of *BIRC5* (Fig. 5e), which resulted in significant enhancement of the FCCP and TRAIL dual treatment effect (Fig. 5f; scRNA: 0.43 ± 0.06-fold change to scRNA vehicle; siRNA: 0.23 ± 0.01-fold change to scRNA vehicle, *n* = 3, *P* < 0.05).

## Discussion and conclusions

Inherent in studies of ovarian cancer is the caveat of the disease’s high degree of heterogeneity in presentation. Because aGCTs represent a small proportion of clinical diagnoses under the umbrella term of “ovarian cancer” many clinical treatment paradigms are developed in context of epithelial adenocarcinomas [7], with a limited number of small clinical studies specifically targeting aGCTs [30]. While prognosis following stage I detection is excellent [7], high rates of recurrence of aGCTs necessitates a further evaluation of the molecular characterization of these tumors, which are likely distinct from epithelial carcinomas [7]. These data represent a characterization of the metabolic profile of KGN cells as a valuable model for aGCTs, with comparative assays run using epithelial-origin tumor derived cell lines instructive in demonstrating the heterogeneous response to treatments, even at the basic level of cells in vitro.

**Fig. 6:**
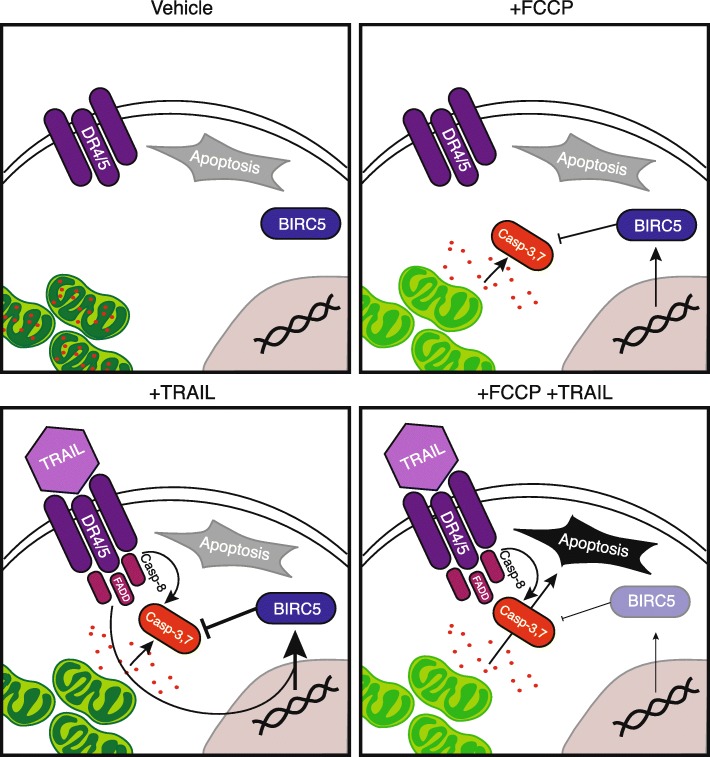
Summary mechanism of Δψ_m_ depolarization enhances TRAIL-induced cell death through inhibition of BIRC5. Proposed mechanism depicting the interplay of Δψ_m_ and TRAIL resulting in increased induction of apoptosis in KGN cells. In viable and metabolically active cells, mitochondria (green) retain cytochrome c (red), however upon depolarization by either FCCP, or TRAIL (violet) binding with its receptors DR4/5 (purple), cytochrome c is released into the cytosol. In response to TRAIL activation of the DR4/5 intracellular death domain (DD) to form FADD and activate the pro-apoptotic caspase cascade, an upregulation of the inhibition of apoptosis protein family member, BIRC5 (blue), is induced, maintaining cell survival. In cells pretreated with FCCP, or those following a knockdown of *BIRC5* expression, pro-apoptotic signals overcome pro-survival signaling to induce apoptosis, suggesting an essential role for Δψ_m_ in the opposition of pro-apoptotic signals

KGN cells were found to be primarily glycolytic, in both short-term responses to ATP production as well as over longer proliferation assays utilizing inhibitors against glycolysis, oxidative phosphorylation, or Δψ_m_**.** Treatment with FCCP was surprisingly effective in inhibiting proliferation in otherwise glycolytic cells, implicating a role for Δψ_m_ outside of metabolism. In fact, pretreatment with FCCP, a proton ionophore which uncouples oxidation from phosphorylation within mitochondria via export of H^+^ ions [27], sensitized cells to TRAIL or cisplatin, more effectively than treatment with oligomycin A, which specifically inhibits oxidative phosphorylation by blocking the F0 subunit of ATP synthase [31]. We confirmed dual treatment was inducing apoptosis, rather than slowing proliferation rates, through Annexin V labeling, with significant increases in apoptosis occurring only in cells treated with both FCCP and TRAIL in combination. When evaluating cytochrome c as an upstream mediator of apoptotic induction, we found significantly higher rates of cytochrome c release in both the FCCP single treatment and dual treated cells, suggesting FCCP treatment is sensitizing cells to further induction via uncoupling Δψ_m_. Interestingly, the Δψ_m_ probe JC-1 indicated that both FCCP and TRAIL single treatments were each sufficient to depolarize Δψ_m_, which indicates a balance between extrinsic signaling, as TRAIL alone is insufficient to induce cell death, and intrinsic regulation, as FCCP alone is sufficient to decrease cell proliferation but not to induce apoptosis. Additionally, we found the IAP, BIRC5, to be essential in this balance, as treatment with TRAIL significantly increased *BIRC5* expression, however this response was lost upon pretreatment with FCCP. A knockdown of *BIRC5* prior to treatment further sensitized cells to apoptotic induction. These data indicate an essential role for both Δψ_m_ and *BIRC5* in KGN cell response to TRAIL as summarized in Fig. 6. These data present the first molecular characterization of Δψ_m_ in KGN cells and its essential role in the inhibition of pro-apoptotic signaling, independent of a metabolic role. Such findings suggest the development of new therapeutic strategies targeting Δψ_m_ to more specifically treat aGCTs, in comparison to EOCs. Although there is limited clinical data focused solely on aGCTs, others have also described glycolytic signatures [32], with promising initial studies in mural models of GCTs [33], however the GCT field remains sparse, in comparison to EOCs. The availability of the KGN cell line to model aGCTs in vitro provides an important opportunity to characterize new basic research strategies, such as deciphering the role played by mitochondria as a hub of cellular signaling in response to cytotoxic treatment, independent of small clinical cohorts. These data indicate a promising new strategy for potential future aGCT-specific treatments, targeting mitochondrial signaling networks in otherwise glycolytic cells.

## Methods

### Cell culture and media conditions

The adult-type human ovarian GCT line, KGN [34], were generously provided by Drs. Nishi and Yanase (Kyushu University, Japan), and maintained in DMEM/Ham’s F12 (Corning) supplemented with 10% FBS (Peak Serum), as described previously [18]. Ovarian epithelial adenocarcinoma cell lines were purchased from ATCC and included: SKOV3, McCoy’s 5a (Gibco) medium supplemented with 10% FBS; OVCAR3, RPMI-1640 (HyClone) supplemented with 0.01 mg ml^− 1^ insulin and 20% FBS; and Kuramochi, RPMI-1640 supplemented with 10% FBS. A summary of previously published reports [35–37] which robustly characterize the EOCs of interest in regard to histological subtype and mutational profiles inclusive of seven common oncogenes is provided as Additional file 1: Table S1. All cells were routinely subcultured and maintained at 37 °C in a humidified atmosphere of 5% CO_2_, 95% air.

### ATP content analysis

Cells were plated as 1 × 10^4^ cells per well in a 96-well plate in cell type specific media. 18 h after plating, culture media was supplemented to include the metabolic inhibitors 2-deoxy-d-glucose (2-DG; Acros Organics) or oligomycin A (Cayman Chemical) to final concentrations as indicated in the figures (0.2% DMSO vehicle). 6 h following treatment, cells were lysed as per the manufacturer’s protocol for the Promega™ CellTiter-Glo™ 2.0 Assay. Total cellular ATP content was calculated using a standard curve of serially diluted ATP (Roche Applied Science) ranging from 0.5 nM to 5.0 μM. Significance was determined between groups via ANOVA, with post hoc Tukey HSD analysis for three experimental replicates, using the One-way ANOVA (ANalysis Of VAriance) with post-hoc Tukey HSD (Honestly Significant Difference) Test Calculator for comparing multiple treatments available at astatsa.com.

### Culture viability via MTS assay

Cells were plated as 1 × 10^4^ cells per well in a 96-well plate in cell-type specific media. For single treatment experiments, 24 h after plating, culture media was supplemented to include metabolic inhibitors 2-DG, oligomycin A, or carbonyl cyanide-*4*-(trifluoromethoxy)phenylhydrazone (FCCP; Tocris) (0.02% DMSO vehicle for oligomycin A and FCCP). For dual treatment experiments, inhibitors were supplemented and cells were preincubated for 1 h at 37 °C, 5% CO_2_, 95% air prior to further treatment with the cytotoxic agents TRAIL (EMD Millipore) or cisplatin (EMD Millipore). Treated plates were incubated for a further 20-h incubation, at which time the Promega™ CellTiter 96™ AQ_ueous_ One Solution Cell Proliferation Assay (MTS) reagent was added to each well and incubated for an additional 3 h at 37 °C, 5% CO_2_, 95% air for color development via formazan production prior to assessment of color change. The samples were normalized to blank media control wells and assayed against vehicle control cells, using Biotek Synergy H1 plate reader. All data are presented as fold-change relative to untreated controls, with significance determined between groups via ANOVA, with post hoc Tukey HSD.

### Flow cytometry analysis of Annexin V and Δψ_m_

Cells were plated as 1.25 × 10^5^ cells per well in a 6-well plate, and 24 h following plating FCCP was added to a final concentration of 5.0 μM and preincubated for 1 hour prior to the addition of 50 ng ml^− 1^ TRAIL, and returned to the incubator for an additional 20-h incubation. Cells were collected and prepared for analysis of apoptosis via binding Annexin V to extracellular phosphatidylserine, or for analysis of Δψ_m_ using the fluorescent dye, JC-1, by flow cytometry. For experiments examining apoptotic induction, additional cells were prepared as an induced necrosis control following incubation with 2 mM H_2_O_2_ for 4 hours prior to labeling. Cells were labeled for 15 min at 37 °C, 5% CO_2_, 95% air per the manufacturer’s recommendation, with the metabolic dye resazurin (Acros Organics) at a final concentration of 500 nM and a FITC-conjugated monoclonal antibody against Annexin V (Invitrogen; clone: VAA-33), and maintained on ice until analysis. Immediately prior to analysis, DAPI (4′,6-Diamidino-2-Phenylindole, Dihydrochloride; Sigma) was added to a final concentration of 200 ng ml^− 1^. All gates for flow cytometry analysis were set utilizing single color stained control samples. For experiments examining Δψ_m_, cell suspensions were stained with 2.0 μM JC-1 (Marker Gene) for 15 min at 37 °C, 5% CO_2_. Stained cells were washed with 1x PBS (Gibco), maintained on ice, and 200 ng ml^− 1^ DAPI (Sigma) was added immediately prior to analysis. All gates for flow cytometry analysis were set utilizing unstained control samples. Analyses were completed using a BD FACSAria III. The JC-1 Red: JC-1 Green ratio was determined.

### Immunocytochemistry of cytochrome c release

Cells were grown on glass coverslips and treated with FCCP and TRAIL, as described above, were fixed in 2% formaldehyde (Electron Microscopy Sciences) and permeabilized in 0.25% Triton-X (Sigma). After washing, coverslips were blocked in 5% BSA (Jackson Immuno Research Labs) for 1 hour at room temperature (22 °C), and washed with PBS. A mouse monoclonal antibody to the beta subunit of ATP synthase (ATBP; Abcam; ab14730, 1 μg ml^− 1^), a mitochondrial protein, and a rabbit oligoclonal antibody to cytochrome c (Invitrogen; 710,627, 5 μg ml^− 1^) were diluted in 1% BSA and coverslips were incubated overnight at 4 °C. After washing with PBS, species specific secondary antibodies (Goat anti-mouse Alexa488, Invitrogen; Goat anti-rabbit Alexa568, Invitrogen) were diluted 1:500 in 1% BSA and coverslips were incubated for 1 hour at room temperature (22 °C). After washing, cells were incubated with 200 ng ml^− 1^ DAPI for 10 min at room temperature (22 °C), and mounted onto slides using Prolong Gold (Life Technologies). Fields of view were randomly selected and focused at the nuclear plane using DAPI labeling, (*n* = 98–250 cells per condition), imaged, and scored according to localization of cytochrome c as either densely colocalized with ATPB-positive mitochondria or diffuse localization in the cytoplasm, indicative of release from mitochondria. Counts were normalized to total number of nuclei, with representative images presented.

### Gene expression analysis

For all gene expression analyses, cultures were treated as described above, and total RNA was isolated using RNazol®RT (Molecular Research Center). One microgram of isolated RNA per sample was treated with DnaseI (Thermo Fisher Scientific) to remove potential genomic DNA contamination, followed by first strand cDNA synthesis using a RevertAid RT kit (Thermo Fisher Scientific). Quantitative analysis of *Multi-Drug Resistance Gene 1* (*MDR1,* also referred to as *P-gp, ABCB1,* or *CD243;* assay ID: Hs00184500_m1), *Death Receptor 4 (DR4,* also referred to as *TRAILR1* or *TNFRSF10A*; assay ID: Hs00269492_m1), *Death Receptor 5* (*DR,* also referred to as *TRAILR2* or *TNFRSF10B*; assay ID: Hs00366278_m1), and *BIRC5* (assay ID: Hs00977611_g1) expression was completed using TaqMan gene expression assays (Applied Biosystems) with the reference gene *beta-2-microglobulin* (*B2M*; assay ID: Hs99999907_m1) using TaqMan™ Fast Advanced Master Mix (Life Technoliges) and a StepOnePlus™ Real-Time PCR System (Applied Biosystems), under parameters recommended by the manufacturer for TaqMan gene expression assays. In brief, each reaction was run with 250 nM of 6-FAM™ dye-labeled TaqMan® MGB probe for each gene of interest, 250 nM of 6-VIC® dye-labeled TaqMan MGB probe for *B2M,* and 50 ng of synthesized cDNA in technical duplicates for 40 cycles at an annealing temperature of 60C using fast ramp rate experimental parameters.

### *BIRC5* knockdown

KGN cells were reverse transfected using Lipofectamine LTX with a commercially available dicer-substrate siRNA construct against *BIRC5* (IDT; Design ID: hs.Ri.BIRC5.13.1) or non-targeting control (IDT; Negative Control SdiRNA # 51–01–14-03) at time of plating, and the culture viability was assessed by MTS assay as previously described. *BIRC5* mRNA knockdown was confirmed by qRT-PCR, as described above, 48 h following transfection.

## Additional file

Additional file 1: **Table S1**. Molecular characterization of epithelial ovarian cancer cell lines, SKOV3, Kuramochi, and OVCAR3. To provide additional detail regarding the epithelial ovarian cancer cell lines used in this study, relevant studies were reviewed in regard to the histological subtype and known mutation profile for SKOV3, Kuramochi, and OVCAR3 cells. The seven presented genes, *TP53, BRCA1, BRCA2, PIK3CA, PTEN, KRAS, BRAF, and ARID1A* are frequently reported with mutation in ovarian cancer. Y indicates reported mutation; N indicates no reported mutation. (XLSX 33 kb)
